# Quantification of reaction time in an Arctic population

**DOI:** 10.3389/fspor.2026.1845818

**Published:** 2026-09-24

**Authors:** Jacob Bjerg, Mads Blak Rønberg, Martin G. Jørgensen, Stig Andersen

**Affiliations:** 1 Department of Geriatric Medicine, Aalborg University Hospital, Aalborg, Denmark; 2 Department of Clinical Medicine, Aalborg University, Aalborg, Denmark; 3 Greenland Centre for Health Research, Ilisimatusarfik, University of Greenland, Aalborg, Greenland

**Keywords:** Arctic environment, greenlanders, lifespan, reaction time, real-life perturbations

## Abstract

**Background:**

Greenland is an Arctic environment with snow and ice on uneven terrain posing lifelong physical challenges to people in Greenland. In addition, Greenlanders are a distinct ethnic group. This led us to (1) quantify reaction time among Greenlanders (2) hypothesising a faster reaction time compared to previous findings among e.g., Scandinavians.

**Methods:**

Data were collected using a portable device, the Nintendo Wii Balance Board, with the FysioMeter software validated for estimating reaction time. Participants were healthy Greenlanders living in West Greenland, who filled in a questionnaire on physical activity, disease, prescription medicine, alcohol, and smoking habits. Reaction time was measured for dominant and non-dominant upper and lower extremities.

**Results:**

The 72 women and 42 men participating had a mean age of 50 years, range 20 to 83 years. The age-related increase in reaction time was around 6 milliseconds for each year of advancing age (*p* < 0.001). Reaction time did not differ between dominant and non-dominant upper (*p* = 0.7) or lower (*p* = 0.5) extremities. While the crude comparison showed that age, medication, physical activity and body proportions influenced reaction time, only age (*p* < 0.001–0.055) and height (*p* = 0.002-0.044) remained important in the adjusted analysis.

**Discussion:**

This first study of reaction time in Greenlanders found patterns similar to those observed in e.g., Scandinavians. Interestingly, Greenlanders did not demonstrate faster reaction times despite lifelong exposure to physically demanding and unstable terrain. This finding may be relevant for future research on motor adaptation and environmental influences on balance control.

## 1 Introduction

Reaction time refers to the interval between the perception of a stimulus such as a trip or slip, and the initiation of a physical response to prevent falls (1). Reaction time involves both sensory processing and motor execution, and neuromuscular control and the speed of central processing influence the ability to respond to sudden balance threats (1, 2). Delayed reaction time can prevent timely corrective movements and increase the risk of falls, which is important for active living and critical in older adults as ageing negatively affects the somatosensory processing and the muscular systems (3).

Greenland is an Arctic environment with snow and ice on uneven terrain, and traditionally Greenlanders relied on walking, sledges, and kayaks, methods still influential today. Walking on the slippery, rugged surface requires constant postural adjustments to maintain stability, and Greenlanders’ active living support experience due to environmental differences by exposure to repeated, external unpredictable physical challenges requiring reactions to regain postural stability (4).

Slower reaction times are predictive of future falls and serious injury, and reaction time tests are increasingly used as part of fall risk screening tools (5, 6). Falls among older adults are critical as 30%–50% of all falls among older people require medical attention (7) with an impact on health (8). Limited data exist on the prevalence of falls among Greenlanders (9), while data on reaction time in Arctic people is lacking. A small and portable solution has been developed and validated for objective assessment of reaction time using the Nintendo Wii Balance Board (WBB) (10). Moreover, parallel data in Scandinavian population are available, as our group previously assessed reaction time across the lifespan using the same measurement equipment and protocol (11).

We explored reaction time among Greenlanders, and we hypothesised that Greenlanders would have a faster reaction time than data reported by a study with a Scandinavian population. In addition, we hypothesised that the reaction time among Greenlanders would increase with age and differ with sex.

## Methods

### Setting

Greenland is the world's largest island with challenging terrain. Deep fjords and valleys were shaped by glacial activity into jagged terrain of steep slopes, loose rocks, and uneven ground as visualised in Supplementary Figure S1. The Arctic climate has restricted the construction of roads to a total of less than 400 kilometres, all within towns (12). The harsh climate further complicates movement, and Figure 1 presents the terrain that challenges outdoor mobility for many Greenlanders. Hunting and fishing serve both as livelihood and for recreational purposes and are a cause of physical training.

**Figure 1:**
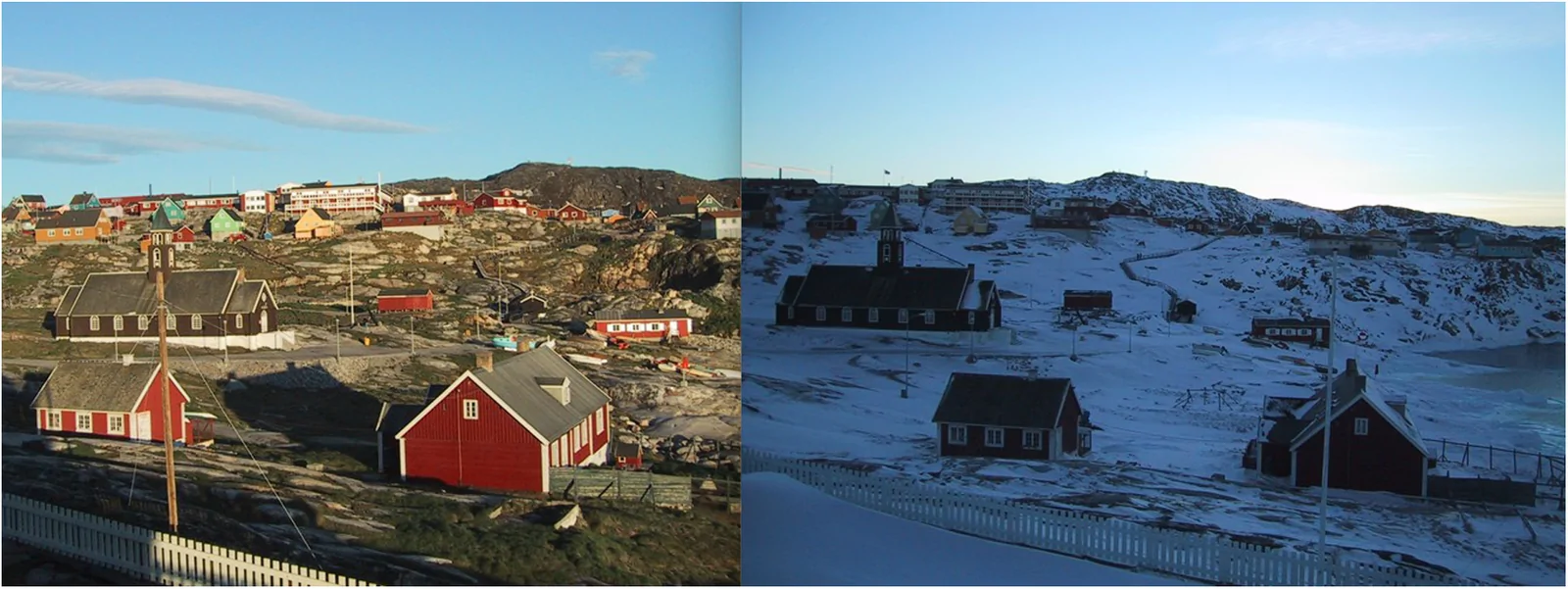
Two parallel photos of the same spot during summer (left) and winter (right) seasons showing the rugged, uneven terrain of Greenland covered by snow and ice during winter. Walking is common in Greenland and active living causes exposure to repeated, external unpredictable physical challenges requiring rapid reactions to regain postural stability.

### Study design and participants

The design was cross-sectional, and the study was performed in the autumn of 2023 in the two major towns, Nuuk and Sisimiut. The authors (JB, MBR) were trained with the test equipment prior to collecting the data. Testing for reaction time was conducted by a single examiner for all participants (JB). The measurement protocol used was similar to the one previously used by our group examining a Scandinavian population and described in detail previously (11).

Recruitment was based on feasibility and the hospitals in Nuuk and Sisimiut hosted most of the data collections with the addition of care homes and public spaces such as the library, mall, and cafés. In addition, invitations were sent by mail to a random sample of the population based on central registration of all inhabitants by residence with additional recruitment using social media, flyers, and among healthcare staff.

We included healthy adults aged 20 years or above with at least one parent born in Greenland. Exclusion criteria were identical to those of the study of Scandinavians (11), and they were cognitive impairment as being unable to state the name of the city of investigation or the present year, or physical limitations as being unable to stand unsupported for 30 s, suffering from neuromuscular disease such as Parkinson's Disease or Myasthenia Gravis, stroke sequelae, severe neuropathy, musculoskeletal disease such as fracture within the last 6 months, alloplastic in the last two years, or muscular dystrophy. The participants stepping height had to exceed 5 cm.

### Questionnaire and measurements

Participants answered a questionnaire on hand and leg dominance, background information on participant and parents’ place of birth, age, medication use, smoking habits, alcohol intake, and physical activity. The questionnaire was available in Greenlandic (kalaallisut) and Danish, and the participants filled out the questionnaire in the language chosen by the participant. Questionnaires were subsequently checked by an interviewer. Though most participants spoke Danish, a local interpreter supported filling in the questionnaire and provided instructions for the tests when needed.

Height and weight were measured prior to measurement procedures. Reaction time measurements were performed using the Wii Balance Board (WBB) (Nintendo, Kyoto, Japan). This is a 5 × 30 × 50 cm portable force plate with four uniaxial vertical strain gauge transducers, positioned at each corner, which collected data at approximately 100 Hz. Data was transmitted via Bluetooth to a PC and processed with Fysiometer® software (Aalborg, Denmark) as described in detail previously (10). Procedures, training and equipment were identical to the study on Scandinavians.

RT was measured for all four limbs in each participant. A computer screen displaying a virtual WBB was positioned on a table 80–100 cm in front of the participant. At test initiation, a green visual stimulus appeared. Participants were instructed to tap the board promptly. Upon tapping, the internal timer ceased, recording the reaction time in milliseconds from visual stimulus onset to board contact. The visual stimulus pointed at the left or right side of the board in random order. Each attempt began immediately after the previous one with a random delay of 1–4 s until a total of seven assessments were performed. This was performed for both upper and lower extremities.

The first six assessments comprised three attempts per side (left and right). The seventh attempt served as a dummy to prevent the participant from guessing the side of the subsequent stimulus. The full seven-attempt session was stopped and repeated if the participant lost focus on the screen, missed the board during hitting, or used the non-designated side to hit the board.

For lower limb reaction time measurement, the WBB was placed on the floor 5 cm in front of the feet positioned shoulder width apart. For upper limb reaction time, the WBB was placed on a table with the participant seated in a chair and hands resting on the table 5 cm in front of the WBB.

All measurements were done in a single session lasting approximately 35 min.

The study was approved by the Ethical Committee for Greenland (2023-20980) and participants provided written informed consent.

### Statistics

No *a priori* sample size calculation was performed, as the study was exploratory and no prior reaction time data from Greenlandic populations were available to inform effect size estimates. Baseline characteristics were presented using frequencies. QQ-plots on the overall dataset were assessed to evaluate if the outcome variables were sufficiently consistent to allow for the statistical analysis. Data followed the normal distribution, and continuous variables were presented as means with 95% confidence intervals. Reaction times were presented as mean and standard deviation (SD) to support future use of data for power calculations. Differences between dominant and non-dominant sides were compared using paired sample t-test and correlations were tested using Pearson's correlation as data followed the normal distribution. The importance of age, sex, medication use, physical activity, smoking, and alcohol were first tested using univariate linear regression models.

Multivariable linear regression was subsequently used because hand and leg reaction times were analysed as separate continuous outcome variables. Separate models were specified for each outcome to estimate the independent associations of participant characteristics with reaction time. Covariates were selected *a priori* based on biological plausibility and previous evidence with age (years), sex (male/female), height (centimetres, cm) or weight (kilograms, kg), medication use (number of prescription drugs) and physical activity (as a composite of work and leisure time, hours/week) entered simultaneously to account for potential confounding and interrelationships among the explanatory variables. Using the same model specification for both upper and lower extremity outcomes allowed for comparison of the adjusted associations while acknowledging that hand and leg reaction times represent distinct physiological responses. Prior to model fitting, the assumptions underlying linear regression were evaluated. Normality of the model residuals was assessed using histograms and normal Q–Q plots, linearity and homoscedasticity by visual inspection of residual-versus-fitted plots, and multicollinearity using variance inflation factors (VIF), with values <2 considered acceptable. No substantial violations of the model assumptions were identified. Potential outliers were identified by visual inspection of scatterplots and subsequently examined individually for plausibility and data quality. Two participants had reaction times more than two standard deviations above the age-specific average. Following individual review, these values were considered unlikely to reflect the reaction time of neurologically healthy individuals, and upon follow-up one was identified as having cerebral tuberculosis and the other documented inability to follow instructions. Hence, they were excluded from the analyses.

A *p*-value of less than 0.05 was considered statistically significant.

## Results

### Demographics

Participants comprised of 114 Greenlanders (42 males and 72 females). Data were collected from 56 individuals in Nuuk and 58 individuals in Sisimiut. Participant characteristics are listed in Table 1. The age ranged from 20 to 83 years in women and from 23 to 75 years in men with a slightly lower median age among men compared to women (Table 1). Medication use was similar between men and women (*p* = 0.7). Two out of three reported four hours or less of weekly leisure time physical activity (Table 1) with no difference between men and women (leisure time, *p* = 0.2; work, *p* = 0.8).

**Table 1:** Participant characteristics and reaction time in the four extremities by age.

	Men		Women		All	
Number of participants (n)	42		72		114	
Age (years) (mean;SD)	48.5	17.8	50.7	15.5	49.9	16.3
Height (cm) (mean;SD)	170.1	8.2	159.2	7.9	163.5	9.7
Weight (kg) (mean;SD)	86.9	17.8	72.3	17.7	77.7	19.0
BMI (kg/m^2^) (mean;SD)	29.7	5.1	28.4	6.4	28.9	6.0
Smoking (n; %)						
Never	11	26%	12	17%	23	20%
Past	13	31%	31	43%	44	39%
Present	18	43%	29	40%	47	41%
Alcohol (n; %)^a^						
Never	6	14%	11	15%	17	15%
<7 units/week	24	57%	50	70%	74	65%
8–14 units/week	7	17%	6	8%	13	11%
>14 units/week	5	12%	4	6%	9	8%
Medicine (n; %)						
No	25	60%	41	57%	66	58%
Yes	17	40%	31	43%	48	42%
Number of drugs (n; %)						
0	25	60%	41	57%	66	58%
1–4	14	33%	28	39%	42	37%
5+	3	7%	3	4%	6	5%
Work activity level (n; %)						
Sedentary	8	19%	10	14%	18	16%
Up-and-about	8	19%	24	33%	32	28%
Active	6	14%	17	24%	23	20%
Hard manual labor	11	26%	12	17%	23	20%
No work	9	21%	9	12%	18	16%
Leisure time activity (n; %)						
Light strain <2h/week	6	14%	15	21%	21	18%
Some, 2–4 h/week	21	50%	32	44%	53	47%
Some, >4 h/week	9	22%	16	22%	25	22%
Heavy, >4 h/week	6	14%	9	13%	15	13%
Reaction time (mean; SD)						
Upper Extremity						
Dominant						
20–39y	564	147	562	108	563	126
40–59y	618	96	689	146	671	137
60+y	726	199	778	202	758	200
Non-dominant						
20–39y	547	103	558	95	553	97
40–59y	578	104	663	120	641	121
60+y	711	193	820	350	778	301
Lower extremity						
Dominant						
20–39y	766	98	775	202	771	159
40–59y	949	343	928	163	934	220
60+y	906	179	1,053	243	1,002	231
Non-dominant						
20–39y	759	90	809	179	785	144
40–59y	948	321	904	184	916	224
60+y	915	180	1,032	237	992	223

a Data missing in 1 participant.

### Reaction time

Table 1 lists reaction time for upper and lower extremities split by sex. Reaction time did not differ between dominant and non-dominant upper (*p* = 0.7) or lower (*p* = 0.5) extremities, and the correlation analysis showed a rise in reaction time with age (r^2^ 0.51/0.48 for dominant/non-dominant upper extremities; r^2^ 0.44/0.41 for dominant/non-dominant upper extremities; p_both_ <0.001).

Figure 2 illustrates the age-related increase in reaction time (beta), which was around 6 milliseconds for each year of advancing age: beta for dominant upper extremity 5.5 milliseconds, non-dominant upper extremity 6.3, dominant lower extremity 6.4, and non-dominant lower extremity 5.6 milliseconds.

**Figure 2:**
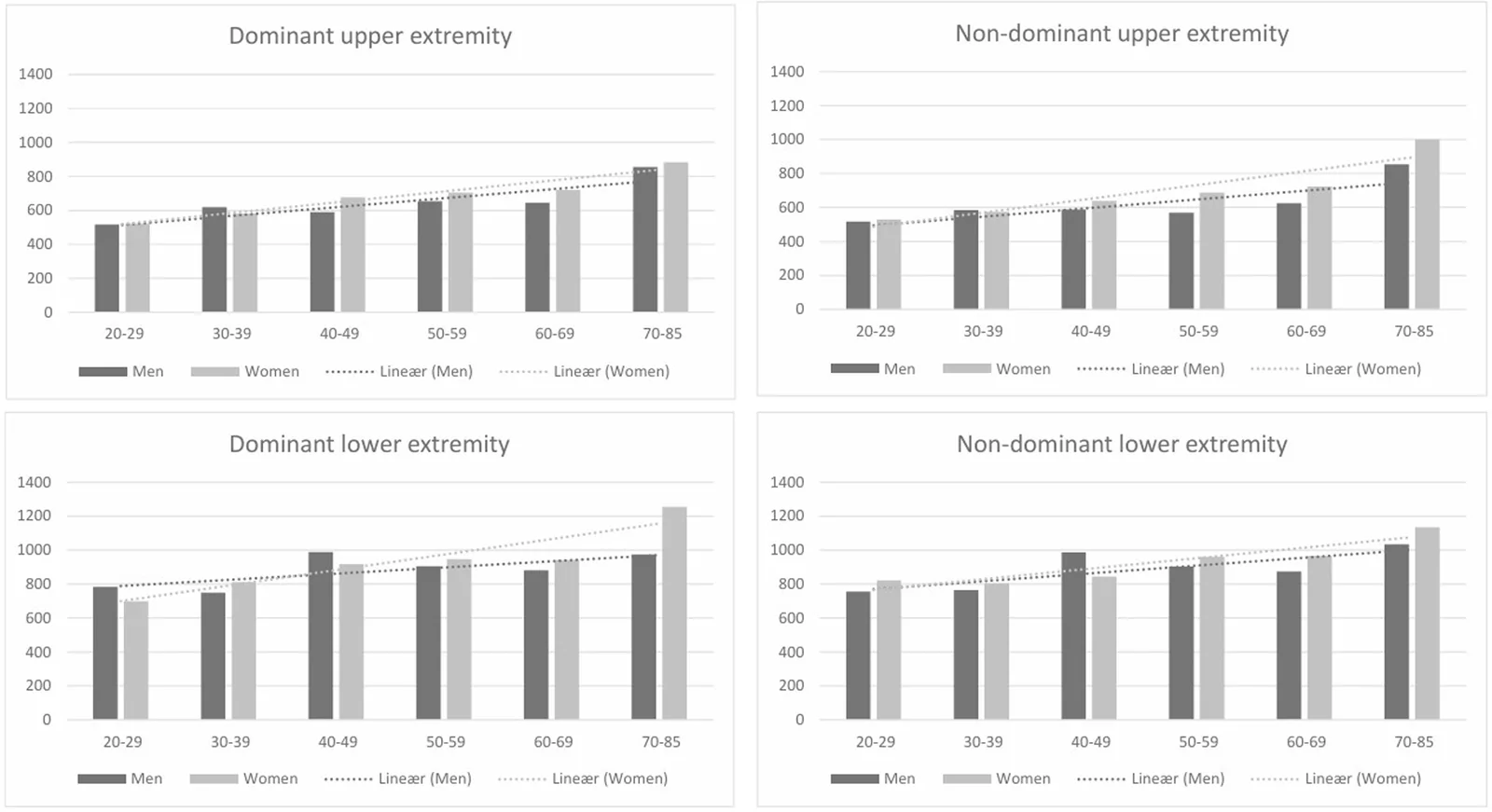
Average reaction times for upper extremities (upper bars), lower extremities (lower bars) with dominant extremity (left) and non-dominant extremity (right) among men and women aged 20−83 years living in Greenland. Broken lines illustrate the increase in reaction time with age.

Regression analysis of the influence of participant characteristics on reaction time is listed in Table 2. While the crude comparison showed that age, medication, physical activity and body proportions had an influence on reaction time, only age and height were important in the adjusted analysis, and the association between height and reaction time was seen for both upper and lower limbs. Sex did not influence reaction time in neither the crude nor the adjusted analysis.

**Table 2:** Linear regression analyses of the impact of variables to reaction speed.

	Lower extremity				Upper extremity			
	Dominant		Non-dominant		Dominant		Non-dominant	
	P		P		P		P	
Univariate								
Age	<0.001		<0.001		<0.001		<0.001	
Sex	0.13		0.17		0.13		0.071	
Height	<0.001		<0.001		<0.001		<0.001	
Weight	0.006		0.015		0.037		0.015	
Medication	0.013		0.006		0.001		0.006	
Physical activity	0.004		0.001		0.011		0.017	

	Beta	P	Beta	P	Beta	P	Beta	P
Multivariate								
Age	0.33	0.001	0.27	0.004	0.43	<0.001	0.38	<0.001
Sex	-	0.54	-	0.24	-	0.92	-	0.96
Height	−0.24	0.012	−0.30	0.002	−0.18	0.044	−0.24	0.010
Weight	-	0.37	-	0.71	-	0.44	-	0.47
Medication	-	0.48	-	0.46	-	0.18	-	0.53
Physical activity	-	0.23	−0.16	0.076	-	0.98	-	0.91

	B (95% CI)		B (95% CI)		B (95% CI)		B (95% CI)	
Age	4.9 (2.6; 7.6)		3.7 (1.2; 6.4)		4.6 (2.7; 6.5)		4.7 (2.4; 6.9)	
Height	−5.7(−10; −1.3)		−6.7 (−11; −2.6)		−3.2 (−6.3; −0.01)		−4.8 (−8.5; −1.1)	
Physical activity			−20 (−44; 4.4)					

" - " *p* >0.1.

B (95% CI), Beta coefficients with 95% confidence intervals.

The differences from an external historical Scandinavian dataset are listed in Supplementary Table S1 (11). Reaction times were numerically higher (i.e., slower) in Greenlanders across all age groups and extremities.

## Discussion

This first study of reaction time among Arctic Inuit included 114 Greenlanders measuring reaction time using the validated portable method. The hypothesis of an increased reaction time with age was confirmed with similar age-related increases for all extremities, while the hypothesised sex difference could not be confirmed. Interestingly, the findings disagreed with the hypothesis of faster reaction times among this population trained by the real-life repeated, external unpredictable perturbations of Arctic living.

Greenlandic Inuit showed genetic signatures of adaptation to living in the Arctic environment (13), which is characterised by a physically demanding landscape, with cold seasons bringing icy terrain and deep snow that require specialized physical skills for outdoor activity. This is further supported by evidence of high levels of physical activity among Greenlanders shaped by living conditions of hunters prior to the modernisation of the society of Greenland. This evidence includes a low occurrence of overweight and diabetes (14, 15), relatively low occurrence of ischemic heart disease (16), and an age distribution of ECG signs of left ventricular hypertrophy supporting that vigorous physical activity was a prerequisite for life in the Greenland hunter population prior to westernisation (16, 17).

Greenland is the Earth's largest island, and even today no two towns are interconnected by road or rail, and the total road network spans less than 400 kilometres. The absence of roads is a practical adaptation to the terrain and extreme weather conditions. Traditionally, Greenland Inuit adapted to this environment by walking and by using sleds and kayaks for transportation. This tradition influences everyday transport in present day Greenland, which is often by foot, and leisure activity in Greenland is dominated by outdoor activity. Hence, walking in the rugged and slippery terrain challenges Greenlanders’ balance by imposing repeated, external unpredictable perturbations triggering reactions to regain postural stability (4). Okubo and colleagues found improved balance recovery responses to trips and slips when exposed to repeated unpredictable perturbations (18), and stepping interventions significantly reduced laboratory-induced falls and improved simple and choice stepping reaction time (19). Our finding of slower reaction time in Greenlanders may suggest an influence of other mechanisms to cope with the active living in Greenland of exposure to repeated external unpredictable physical challenges. It may be speculated that the proper reactions among Greenlanders to regain postural stability differs from that of other populations, who rely on rapid reaction times (4). The mechanisms underlying the observed differences in reaction time remain uncertain.

Though the definition of reaction time is simple, the physiology is not. Reaction time involves detection and discrimination of a stimulus, decision making regarding the appropriate response, planning and organization of the response, and finally response initiation and execution (1). These multiple steps leave room for modifying reaction responses as illustrated by the findings that reaction times are influenced by prior experience (20), that external conditions affect the simple reaction time (21), and that the complexity of task increases reaction time. It may be speculated that experiencing repeated, external unpredictable real-life perturbations by outdoor activities in Greenland provides different or more complex inputs that have formed adaptation among Greenlanders. Hence, Greenlanders may have adapted and use other stimuli than visual, and they may respond faster to tactile or auditive stimuli. Thus, the measurement of reaction time may seem straightforward, as it involves recording the amount of time that elapses between the presentation of a stimulus and the initiation of a response (1), but a reaction is heavily dependent on the stimulus and training.

The association between height and reaction time could partly reflect longer neural conduction distances in taller individuals, a mechanism proposed in other populations. Also, the reaction time difference between upper and lower extremities was relatively similar across age groups. This pattern is compatible with a contribution from longer neural conduction distances for lower-limb responses, a delay expected to be relatively constant across age groups. Thus, conduction distance may represent one biologically plausible explanation, but the mechanism remains speculative and was not directly assessed in the present study, and these mechanisms represent hypotheses for future research.

Real-life experience affecting reaction time is likely to influence the direction and force of a response, and task specific training associated with a reduced risk of falls in real life (22). Falls among Greenlanders was described in a questionnaire survey among Greenlanders in the capital Nuuk, and the fraction reporting falls within the previous year was 44%, 61%, and 22% when in their 50-ties, 60-ties, and 70-ties (9). These falls rates are clearly higher compared to those reported among other populations (8). Also, the lower number of falls among those aged 70 + years compared to younger people differs from findings in other populations (9). These discrepancies may support the notion of Arctic terrain for outdoor activities make older people in Greenland stay indoors. However, this study was based on a limited number of respondents and was confined to the capital Nuuk. Thus, training balance recovery with unpredictable perturbations may still be beneficial to fall avoidance in everyday life also in Greenland.

An observed difference in frequency of fall-related injuries between Greenlandic children and children visiting, unfamiliar with the terrain (supplementary S2), sparked the hypothesis that the early-life exposure to the Greenlandic environment may contribute to motor adaptation. While adaptation to Arctic environmental conditions and lifelong exposure to uneven terrain may represent plausible hypotheses, these factors were not directly assessed in the present study and require investigation in studies specifically designed to evaluate balance, gait, postural responses, and sensory integration. Also, comparisons between the Greenlandic and the external dataset of Scandinavian populations should be interpreted with caution, as only the measurement protocol used was similar between the parallel studies. The number of participants is a limitation of the study. Still, the age-related changes are in keeping with those previously reported (11) and the lack of a sex difference has also been found among other people with an active lifestyle (21). These similarities with our findings support the validity of the present study. The use of a visual stimuli is a limitation that warrants further studies using other paths of stimulation to corroborate the findings. Because participation was voluntary and recruitment was conducted through community and healthcare settings, selection bias cannot be excluded. Still, recruitment both in the capital city and in a smaller city as well as recruitment through multiple community-based settings broadened participation. The addition of data among inhabitants in small villages and settlements was not feasible for the present study for practical reasons, but it remains relevant to corroborate the findings. Finally, the number of comparisons increases the risk of false-positive findings, though the findings were similar across extremities.

In conclusion, this is the first study of reaction time among Arctic inhabitants. We found age and sex differences similar to findings among other people. Interestingly, Greenlanders did not demonstrate faster reaction times despite lifelong exposure to physically demanding and unstable terrain. The mechanisms underlying the observed differences in reaction time remain uncertain. Future studies on motor adaptation and environmental influences on balance control should investigate balance, gait, postural responses, and sensory integration to clarify potential mechanisms compensating for the slower reaction times.

## Data Availability

The raw data supporting the conclusions of this article will be made available by the authors, without undue reservation.
