# Supplementary material for: Quantification of reaction time in an Arctic population
Source: Front Sports Act Living. 2026 Sep 24;8:1845818. doi: 10.3389/fspor.2026.1845818 (PMC13648249; doi:10.3389/fspor.2026.1845818)
Supplement: Supplementary file 1 [file Supplementaryfile1.docx]

Supplementary

Figure S2

Topographic atlas of Greenland (left) and Denmark (right)

<https://da-dk.topographic-map.com/map-nvw951/Danmark/?center=55.57834%2C12.37061>
